# First Isolation and Characterization of Three Strains of Porcine Sapelovirus in Yunnan Province, China

**DOI:** 10.3390/v17040505

**Published:** 2025-03-31

**Authors:** Pei Zhu, Zhanhong Li, Zhuoran Li, Li Meng, Peng Liu, Xiutao Sun, Qi Yang, Jianling Song

**Affiliations:** 1 Yunnan Tropical and Subtropical Animal Virus Diseases Laboratory, Yunnan Animal Science and Veterinary Institute, Kunming 650224, China; zpcau@sina.com (P.Z.); dy081lzh@163.com (Z.L.); lizhu-oran85@126.com (Z.L.); 2 Key Laboratory of Transboundary Animal Diseases Prevention and Control (Co-Construction by Ministry and Province), Yunnan Animal Science and Veterinary Institute, Kunming 650224, China; 3 Honghe Preventive and Control Center for Animal Diseases, Mengzi 661199, China; 4 Mile Preventive and Control Center for Animal Diseases, Mile 652300, China

**Keywords:** porcine sapelovirus, isolation, characterization, phylogenetic analyses, recombination, Yunnan Province

## Abstract

In order to investigate the causes of swine diarrhea in Yunnan Province, this study was conducted to detect and monitor diarrhea viruses through regular sampling and reverse transcription polymerase chain reaction (RT-PCR). In October 2023, porcine sapelovirus (PSV) was detected in fecal specimens collected from diarrheal pigs in Honghe City, and three strains of PSV were successfully isolated by inoculating them into PK-15 cells; electron microscopy revealed virus particles with diameters of ~32 nm. Next-generation sequencing (NGS) revealed that the PSV isolate genomes ranged from 7480 to 7515 nucleotides in length. Homology analyses indicated that ML-15 and ML-16 showed the highest nucleotide and amino acid identities with the Asian PSV strains, ML-19 showed the highest sequence identities with the Zambia PSV strains, and the VP1 to VP4 genes of the three PSV isolates were in the hypervariable region. Phylogenetic analyses showed that the three PSVs isolated in this study all clustered together with Chinese PSV strains; furthermore, recombination analyses indicated that PSV-ML-19 might be a recombined strain and may have emerged through genetic recombination between the major putative parent strain PSV-21-V and the minor putative parent GER L00798-K11 14-02. This was the first reported instance of the isolation and phylogenetic analyses of the PSV strains in Yunnan Province, which enriched the understanding of Chinese PSV strains and indicated the need to prevent and control PSV; the mutation of the VP1 and 3D genes may also provide an important reference for the development of PSV vaccines.

## 1. Introduction

PSV is a single-positive-strand RNA virus, the smallest known animal RNA virus, which was first identified in England in 1960 [1]. PSV can cause respiratory, intestinal, and reproductive-system-related diseases, polio, and other diseases in pigs of all ages; piglets infected with PSV mainly show symptoms of diarrhea, pneumonia, and poliomyelitis. In China, the first report of PSV originated from a swine farm following an outbreak of porcine encephalomyelitis in 2009 [2]. Molecular and serological investigations revealed that PSV was highly prevalent in Chinese pigs [3]; that co-infections with PSV and other enteric pathogens, such as swine fever virus, porcine teschovirus, porcine epidemic diarrhea virus, and porcine enterovirus, are common [4]; and that the clinical symptoms are complex and difficult to distinguish, which drew the attention of researchers to PSV.

Sapeloviruses belong to the genus Sapelovirus, a newly recognized taxon within the family Picornaviridae. PSV is a microsphere particle without viral vesicle; its average diameter is about 28–30 nm and its genome length is about 7.5 kb, showing a typical picornavirus genome organization as follows: a 5′ untranslated region (UTR), a single large open reading frame (ORF), a 3′-UTR, and a poly (A) tail. The ORF encodes a single polyprotein that is subsequently cleaved into twelve proteins by virus-encoding proteases, including a leader protein, four structural proteins (VP1, VP2, VP3, and VP4), and seven nonstructural functional proteins (2A, 2B, 2C, 3A, 3B, 3C, and 3D) [5]. VP1 is the outermost surface protein of viral particles, induces the production of neutralizing antibodies, contains binding sites for cellular receptors, and is a key protein for the viral invasion of cells; VP1 is also commonly used for genotyping and has proven useful in determining genetic relationships among picornaviruses [6]. PSV was originally isolated from the intestines of diarrheic pigs and was formally named porcine enterovirus 8 (PEV-8) [7]. However, further detailed genetic and phylogenetic analyses have indicated that PEV-8 has a unique gene structure, including L and 2A gene regions, which are genetically distinct from those of other porcine enteroviruses. In 2014, the International Committee on Classification of Viruses (ICTV) officially named it porcine sapelovirus, categorizing it into the genus sapelovirus of the small RNA virus family (picornaviridae). Previously, PSV has been considered to be composed of a single genotype, PSV-1 [8], while Hungarian scholars recently discovered a potential novel genotype, PSV-2 [9], with some studies determining two genotype classifications by analyzing the genetic phylogenetic tree of PSV strains in the database [10].

Porcine sapropavirus reports are on the rise globally. In 1960, Lamont P et al. isolated PSV for the first time from the feces of diarrheic pigs in England [11], and this was then reported in Canada, Japan, Australia, Brazil, Spain, Korea, and other countries [12]. PSV can infect both domestic and wild boars at a high rate [13]. In 2009, Lan et al. reported the first case of PSV infection in China, resulting in pig herds experiencing acute diarrhea, respiratory distress, ataxia, hind limb paralysis, and death [2]. The RT-PCR results for 960 pig fecal samples from 15 large-scale pig farms in eastern China, obtained by Lan Daoliang et al., showed that all 15 farms were infected with PSV, showing a total positive rate of 17.2% [14]. In 2014, Schock et al. reported an outbreak of encephalomyelitis in 3- to 4-week-old piglets caused by PSV infection in the United States [15]. In 2016, a case of widespread herd morbidity caused by PSV infection was also reported in the United States, where approximately 3000 fattening herds exhibited atypical neurological signs, with 20% morbidity and 30% mortality rates [16]. Hayato Harima et al. investigated 147 fecal samples collected in Zambia in 2018 and found a high prevalence of PSV infection in suckling and fattening pigs (36.2% and 94.0%, respectively) [17]. Lisandru Capai et al. investigated 908 pig fecal samples from 16 pig farms in Corsica, France, and determined a PSV detection rate of 62.0% (563/908); PSV was found on almost all of the farms, indicating widespread distribution [4].

PSV can destroy intestinal barrier integrity, thus triggering diarrhea in piglets, and can synergize with a variety of viruses, which can then cause disease. No commercial vaccines or diagnostic products are currently available, making the prevention and control of PSV difficult, as well as threatening the sustainable development of the swine breeding industry.

In this study, we collected diarrheal feces from pig farms in Honghe City, Yunnan Province, China, and diarrheal pathogens, including porcine epidemic diarrhea virus (PEDV), transmissible gastroenteritis virus (TGEV), porcine rotavirus A (PRV-A), porcine deltacoronavirus (PDCoV), PSV, enterovirus G (EV-G), and porcine teschovirus (PTV), were detected using RT-PCR. Three strains of PSV were successfully isolated using laboratory methods, and the viruses were purified, identified, and biologically characterized simultaneously. Next-generation sequencing (NGS) revealed that the genomes of PSV isolates ranged from 7480 to 7515 nucleotides in length. Phylogenetic analyses showed that the three PSVs isolated in this study all clustered together with Chinese PSV strains; furthermore, the recombination analyses indicated that PSV-ML-19 might be a recombined strain and may have emerged through genetic recombination between the major putative parent strain PSV-21-V and the minor putative parent GER L00798-K11 14-02.

This was the first reported instance of PSV isolation in Yunnan Province involving comprehensive phylogenetic analyses of the complete-genome sequences of the isolated strains. The results enrich our understanding of PSV in China and indicate the need to prevent and control PSV in Yunnan herds; the mutation of the VP1 and 3D genes may provide an important reference for the development of PSV vaccines.

## 2. Materials and Methods

### 2.1. Sample Collection, Virus Isolation, and Identification

In December 2023, 30 fecal specimens were collected from diarrheal pigs in Honghe City, Yunnan Province, China. The samples were diluted at a ratio of 1:10 with phosphate-buffered saline (PBS) and then centrifuged at 3000 rpm for 15 min at 4 °C. The total viral RNA/DNA were extracted from 200 µL of suspension liquid using the MiniBEST Viral RNA/DNA Extraction Kit Ver. 5.0 (Takara Bio, Dalian, China), according to the manufacturer’s instructions. The nucleic acids of porcine epidemic diarrhea virus (PEDV), transmissible gastroenteritis virus (TGEV), porcine rotavirus A (PRV-A), porcine deltacoronavirus (PDCoV), and PSV were detected using RT-PCR [1,2], applying the PrimeScriptTM One Step RT-PCR Kit (Takara Bio, Dalian, China). PSV-positive samples were diluted at a ratio of 1:20 with phosphate-buffered saline (PBS) containing penicillin and streptomycin and then centrifuged at 12,000 rpm for 20 min at 4 °C. The suspension was filtered through 0.45 μm and 0.22 μm filters and stored at −80 °C. Porcine kidney (PK-15) cells (the China Center for Type Culture Collection, Wuhan, China) were cultured in 25cm^2^ flasks with Dulbecco’s Modified Eagle’s Medium (DMEM) (Gibco, Invitrogen, CA, USA) containing 8% heat-inactivated fetal bovine serum (FBS) (Gibco), 0.1 mg/mL streptomycin, and 100 U/mL penicillin. Then, volumes of 1mL per 25 cm^2^ of the PSV-positive samples were inoculated for 1 h; 10 mL of maintenance DMEM with 1% FBS, 0.1 mg/mL of streptomycin, and 100 U/mL of penicillin was added, whereas the suspensions were discarded. The cells were cultured at 37 °C in 5% CO_2_. The cytopathic effect (CPE) was monitored daily, and three to five blind passages were performed until an obvious CPE appeared. The infected cells were harvested and subjected to three freeze–thaw cycles, centrifuged at 8000 rpm for 15 min at 4 °C, and blindly passaged for three generations, while the cell lesions were monitored for signs of cytopathic effects (CPEs). When obvious cell lesions appeared, the sample was repeatedly frozen and thawed 3 times at −80 °C, and the supernatant was collected via centrifugation at 8000 rpm for 15 min. The total viral RNA was extracted using the MiniBEST Viral RNA/DNA Extraction Kit Ver. 5.0 (Takara Bio, Dalian, China), according to the manufacturer’s instructions. PSV, PEDV, TGEV, PRV-A, PDCoV, EV-G, and PTV’s nucleic acids were detected using RT-PCR, applying the PrimeScriptTM One Step RT-PCR Kit (Takara Bio, Dalian, China). The reaction mixtures were observed using 1% agarose gels for electrophoresis after amplification, and the expected DNA bands were sequenced by Shanghai Shenggong Bioengineering Co (Shanghai, China).

### 2.2. Virus Purification and TCID50

To purify the isolated viruses, a plaque assay was performed as previously described. PK15 cells were cultured in 12-well plates and inoculated with a 10-fold serially diluted virus (0.4 mL/well) for 1h at 37 °C; then, the virus inoculum was discarded and the plates were washed twice with PBS. The cells were inoculated with 2 mL of MEM supplemented with 1% (*w*/*v*) agarose (Sigma Aldrich, St. Louis, MO, USA) and 1% fetal bovine serum (FBS; Gibco) and were incubated at 37 °C for 96 h. After plaque development, clear and uniform plaques were picked and reinoculated into the PK-15 cell monolayers to harvest the positive clones. The virus clones were successfully obtained after about three rounds of plaque purification. Then, the plates were fixed with paraformaldehyde and stained with crystal violet in order to observe the viral plaques.

The virus titers were measured using 10-fold serial dilutions in PK15 cells that were seeded into 96-well plates and were calculated as 50% tissue culture infectious doses (TCID 50) per mL according to the Reed–Muench method.

### 2.3. Replication Kinetics Analysis

Three 75 cm^2^ monolayers of PK-15, IPEC-J2, ST (swine testis), BHK-21 (Baby Hamster Syrian Kidney), Vero, and MDBK (Madin Darby Bovine Kidney), collected from the China Center for Type Culture Collection, were infected with PSV at a 0.01 multiplicity of infection (MOI). After incubation for 1 h, the cells were washed twice with PBS, and 30 mL of maintenance DMEM medium was added. Following this, 100 µL samples of the cell supernatants were harvested 6 h, 12 h, 24 h, 36 h, 48 h, 60 h, 72 h, 84 h, and 96 h post inoculation. The supernatants were centrifuged at 12,000 rpm for 10 min at 4 °C. The viral titers of samples collected at each time point were determined via viral plaque counting, as described in Section 2.2, and viral proliferation curves were plotted using GraphPad Prism 6 software.

### 2.4. Observation of the Virus Using Transmission Electron Microscopy

PK-15 cells were infected with the isolated strains and cultured. The culture supernatants were collected when the CPE was >80% and were clarified by being centrifuged at 10,000 rpm to remove cell debris. The supernatants were passed through a 0.22 µm filter and centrifuged at 40,000 rpm for 4 h at 4 °C (Beckman, Indianapolis, IN, USA). The resulting pellet was resuspended in DMEM and centrifuged through a 13 mL 20–50% (*w*/*v*) sucrose cushion (Beckman SW55Ti rotor,4 h, 35,000 rpm, 4 °C). The virus particles that formed a white opalescent band at the interface of the sucrose solutions were collected. The cell pellet was resuspended in 1 mL of TNE Buffer (Solarbio, Beijing, China) and stored at 4 °C overnight. The samples were then negatively stained with 2% phosphotungstic acid and observed under a transmission electron microscope (Hitachi TEM system, Japan, HT7800, Tokyo, Japan). To further confirm the viral particles, the cell supernatants were tested using RT-PCR with specific primers, as previously reported.

### 2.5. Virus Genome Sequencing

Total RNA was extracted from 200 µL virus stocks using a viral RNA/DNA extraction kit (Takara Bio, Dalian, China) according to the manufacturer’s instructions, and the extracted viral RNA was quantified using a Nanodrop 2000 (Thermo Scientific, Waltham, MA, USA). Then, the viral RNA was sent to Guangdong MAGIGENE Technology Co., Ltd. (Guangzhou, China), where NGS was conducted. Briefly, the viral RNA was reverse-transcribed with random hexamers using Superscript III Reverse Transcriptase (Life Technologies, Waltham, MA, USA), and then the Klenow fragment polymerase (NEB) was added to synthesize the double-stranded cDNA (dscDNA). The dscDNA was subjected to library construction using the TruSeq DNA Sample Prep Kit v2 DNA (Illumina, San Diego, CA, USA), and then, NGS was performed on the Illumina HiSeq 6000 platform (Illumina). The quality of the raw reads was assessed using Soapnuke [18], and then, adapter sequences, low-quality sequences, and host sequences were removed. After filtration, de novo assembly was performed using Megahit V1.0 [19].

### 2.6. Sequence and Phylogenetic Analysis

A total of 133 PSV genomes (Supplementary Table S1), which were isolated from different countries, were downloaded from GenBank. The nucleotide sequences and the deduced amino acid sequences of the three PSV strains isolated in this study were found to refer to the V13 strain and the PSV2020 strain. The sequences were aligned using the MAFFT software (Version 7.380) [20], the sequences’ identified values were calculated using BioEdit (Version 7.1.3.0) [21], and the sequences’ identified heatmaps were plotted using https://www.bioinformatics.com.cn (last accessed on 10 December 2024), an online platform for data analysis and visualization [22]. The neighbor-joining (NJ) phylogenetic trees of the complete genome and the 3D and VP1 genes were generated via the neighbor-joining method using MEGA v.6.0 [23]. Finally, the Recombination Detection Program 4 (RDP4, Version 4.101) [24] and SimPlot software (Version 3.5.1) [25] were used to evaluate the potential recombination events in the PSV genome sequence.

## 3. Results

### 3.1. Isolation, Identification, and Biological Characteristics of PSV

**Figure 1:**
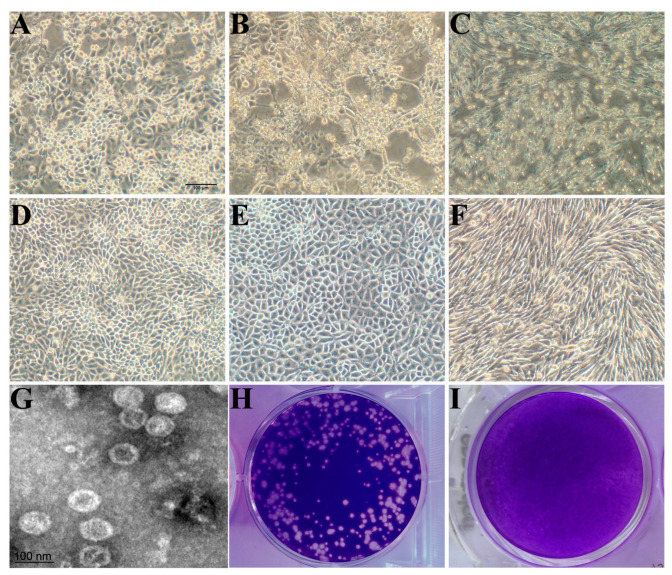
The CPEs, TEM and plaque formation of the PSV-ML-15 strain. (**A**) The CPEs of PSV-ML-15 in IPEC-J2 (scale bar = 100 µm). (**B**) The CPEs of PSV-ML-15 in PK-15 (scale bar = 100 µm). (**C**) The CPEs of PSV-ML-15 in ST (scale bar = 100 µm). (**D**) The cell control of IPEC-J2 (scale bar = 100 µm). (**E**) The cell control of PK-15 (scale bar = 100 µm). (**F**) The cell control of ST (scale bar = 100 µm). (**G**) Transmission electron microscopic image of purified PSV particles. (**H**,**I**) Plaque formation of PSV-ML-15 in PK-15 cells (stained with crystal violet at 72 hpi).

**Figure 2:**
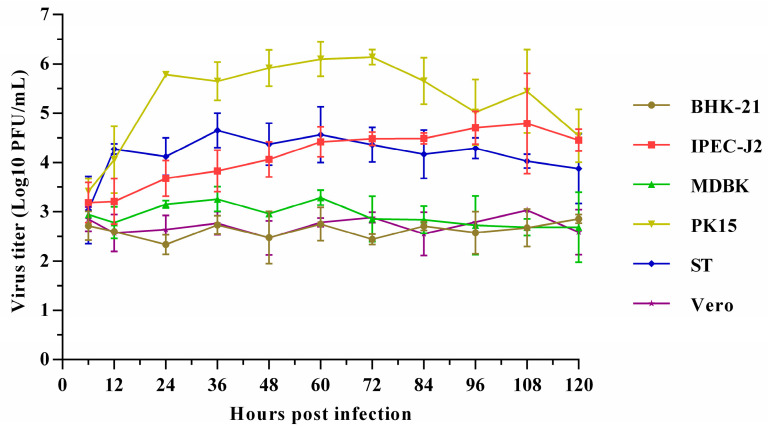
Growth kinetics of PSV-ML-15 in different cell lines with an MOI of 0.01.

Three PSV-positive samples were detected, one of which was coinfected with PEDV, and another with PTV, while the third one was coinfected with both PTV and EV-G. After four blind passage cycles, severe cytopathic effects (CPEs) were induced, with observations of cell rounding, shrinking, and detachment. The virus was purified by plaque purification assay; after three rounds of which, the purified virus developed uniform and clear plaques. PSV was identified from culture supernatants using RT-PCR, and a specific band of about 300 bp was amplified. The sequencing of PCR products and the online comparison with Blast showed that the nucleotide sequence of the isolated strain shared 98% identity with a published PSV strain, which suggests that the isolates are PSV; these isolates were then designated as PSV-ML-15, PSV-ML-16, and PSV-ML-19, respectively. The titer of the purified virus stock was 5.2 × 10^6^ PFU/mL. The viral growth kinetics assay showed that the isolated PSV cannot proliferate on BHK, Vero, or MDBK, while it can successfully proliferate on PK-15, IPEC, and ST (Figure 1), where rapid proliferation was observed at 6 h post infection, reaching a peak of 7.2 × 10^6^ PFU/mL at 72 h; the plateau period began at 84 h and continued until 120 h (Figure 2). The viral titer was 6.7 × 10^6^ PFU/mL at the end of the 120 h sampling. After ultracentrifugation, the plaque-purified viruses were examined under TEM; the results showed that spherical, non-enveloped virus particles of approximately 32 nm in diameter were observed (Figure 1), which is consistent with previous reports. Both the sequencing and TEM results indicate successful virus isolation.

### 3.2. Genome Organization and Homology Analyses

The nearly-whole genomes of the isolated PSV strains were obtained through NGS, and the lengths of the genomes were 7 513 nucleotides (PSV-ML-15, GenBank accession number PV009944), 7 515 nucleotides (PSV-ML-16, GenBank accession number PV009945), and 7 480 nucleotides (PSV-ML-19, GenBank accession number PV009946). Consistent with the previously reported PSV reference strains, the genomes of the three PSV isolates contained 5′ and 3′ untranslated regions (UTRs) and a single large open reading frame (ORF), whose lengths were 7020 nucleotides (ML-15 and ML-16) and 6996 nucleotides (ML-19), respectively, and comprised 12 genes ranging from 66 nt (3B gene) to 1389 nt (3D gene) (Table 1). Notably, the 1D gene of the ML-19 strain was 855 nucleotides in length, making it 24 nt shorter than that of the ML-15 and ML-16 strains, and, correspondingly, the deduced VP1 protein was short at eight amino acids.

At the complete CDS and polyprotein level, ML-15 and ML-16 shared the highest nucleotide (87.76% ± 1.85 and 87.69% ± 1.86) and amino acid (96.05% ± 1.58 and 95.81% ± 1.57) identities with PSV strains isolated from Asian countries, while ML-19 shared the highest nucleotide (87.07% ± 0.72) and amino acid (95.16% ± 0.92) identities with PSV strains isolated from African countries (Zambia strains) (Table 1). The sequence similarities with the American and European PSV strains were smaller than those with the Asian and African strains (Table 1). In detail, ML-15 and ML-16 showed the highest nucleotide sequence identity (91.90%) with the PSV strain QT2013 (KJ463384.1) isolated from China, (Table S1) and ML-19 was most closely related to another Chinese strain, HuN22, with a 90.20% nucleotide identity. At the amino acid level, ML-15 showed the highest sequence identity (98.50%) with three PSV strains (PoSapV_VIRES_HeB04_C1, HuN26, and HuN5) isolated from China, ML-16 showed the highest sequence identity (98.30%) with the PSV strain HuN26 (Table S1), and ML-19 showed the highest sequence identity (98.70%) with two PSV strains (HuN22 and PF92/con/PicV14) (Table S1) isolated from China.

Furthermore, the complete CDS was truncated into 12 single genes and compared with the homologous regions of other representative PSV strains, and then the sequence identity heatmap was plotted. As Table 1 shows, seven ML-15 genes (L, VP1-3, 2A, 2B, and 3A) exhibited high homology with the Asian PSV strains, while the other genes (VP4, 2C, and 3B-D) displayed high homology with the African PSV strains. ML-16 showed high homology with ML-15, except for the 3C gene, which showed the highest identity with American PSV strains (Table 1). As for ML-19, five genes (VP2-4, 2A, and 3A) showed high homology with the Asian PSV strains and six genes (VP1, 2B-C, and 3B-D) showed high homology with the African PSV strains. The L gene showed the highest nucleotide identity with American PSV strains; however, the amino acid sequence was closest to the sequences of Chinese PSV strains (Table 1).

**Figure 3:**
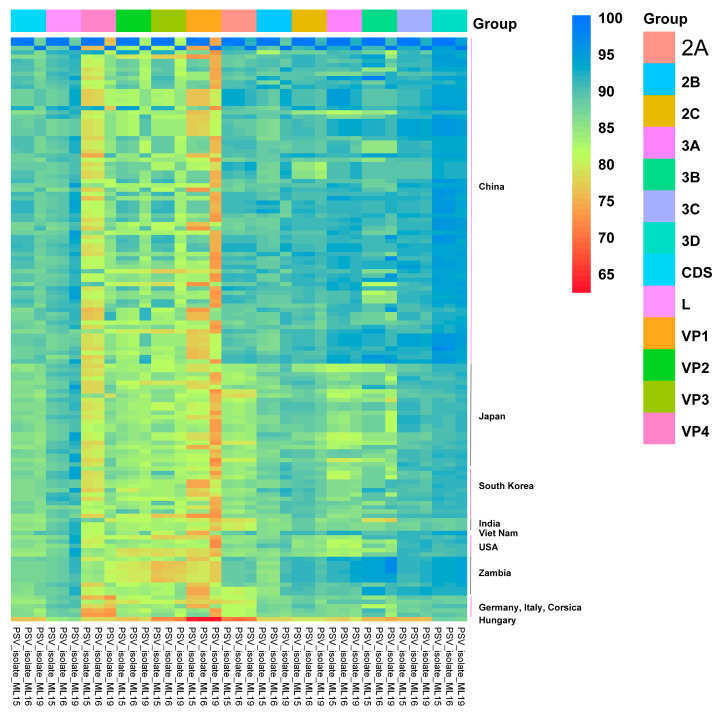
Heatmap of nucleotide identity between PSVs isolated in this study and reference PSV strains. The genomes of 136 PSV strains were divided into 12 sequential genes (L, VP4, VP2, VP3, VP1, 2A to 2C, 3A to 3D), and each gene of reference PSVs was compared with the synonymous gene of the three isolated PSVs in this study; the sequence identity between every pair was measured and represented as a heatmap, scaled such that the minimum inter-sequence identity (62.1%) is displayed as red and the maximum inter-sequence identity (100%) as blue.

**Figure 4:**
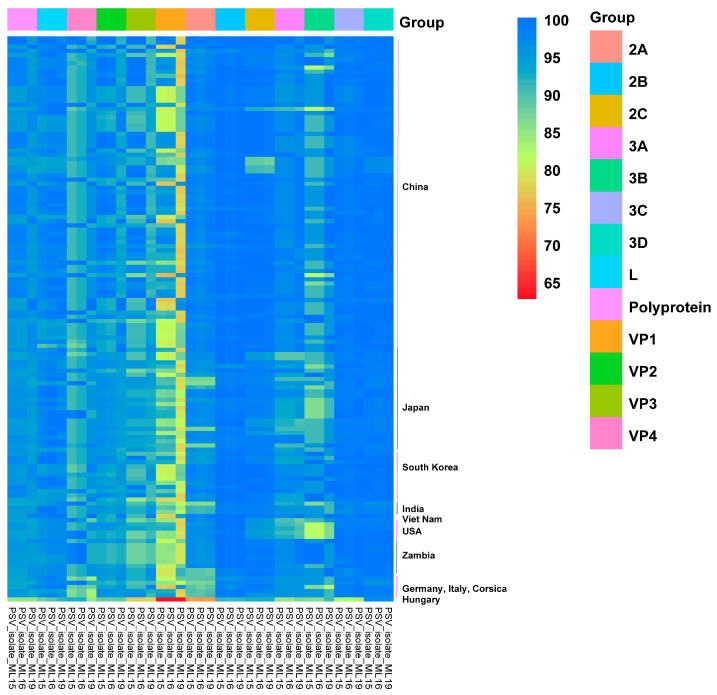
Heatmap of amino acid identity between PSVs isolated in this study and reference PSV strains. The polyproteins of 136 PSV strains were divided into 12 sequential protein (L, VP4, VP2, VP3, VP1, 2A to 2C, 3A to 3D), and each protein of reference PSVs was compared with the synonymous protein of the three isolated PSVs in this study; the sequence identity between every pair was measured and represented as a heatmap, scaled such that the minimum inter-sequence identity (62.5%) is displayed as red and the maximum inter-sequence identity (100%) as blue.

As shown in the identity heatmap, the nucleotide sequences showed more variety than the amino acid sequences (Figure 3 and Figure 4). The VP1 to VP4 genes of the three PSV isolates represented the hypervariable region within the genome (Figure 3 and Figure 4). In detail, the VP1 gene was the most variable, with its highest nucleotide identity being that of the ML-19 VP1 gene, at just 85.40% (PF92/con/PicV14 and PF93/con/PicV15) (Figure 3 and Figure 4, and Table S1). On the contrary, the 3D genes of the three isolates were relatively conserved. The average identity of a nucleotide sequence ranged from 88.34% (ML-19 vs. European PSV strains) to 93.16% (ML-19 vs. Zambian PSV strains), and the average identity of an acid amino sequence ranged from 97.40% (ML-16 vs. European PSV strains) to 99.17% (ML-19 vs. Zambian PSV strains) (Figure 3 and Figure 4, and Table 1). The nucleotide identity heatmap showed that the 2A to 2C and 3A to 3C genes of the three PSV strains isolated in this study were relatively conserved in terms of their identity with the Chinese and Zambian PSV strains, while the identities with the Japanese, South Korean, Indian, American, and European strains were relatively low (Figure 3).

### 3.3. Phylogenetic Analyses of Whole Genome, 3D Gene, and VP1 Gene

**Figure 5:**
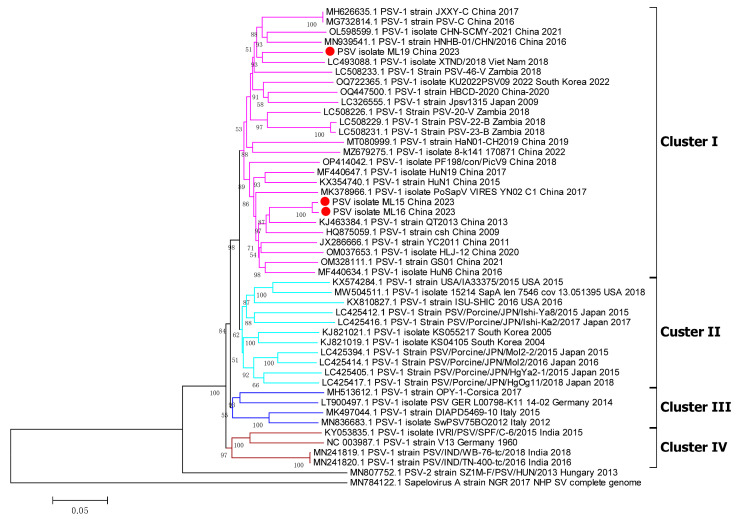
Phylogenetic analysis based on the genomes of PSV strains. The tree was constructed by the neighbor-joining method with MEGA v.6.0. The numbers on the branches are bootstrap values (percent) from 1000 replicates. The three PSV strains isolated in this study are marked with a red dot, and the sequences of other representative PSVs are indicated as ‘GenBank accession number+PSV-1+Strain number+Country+Year’.

**Figure 6:**
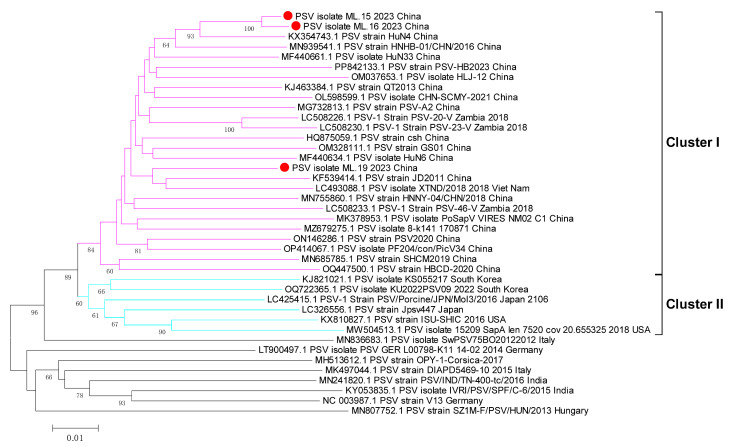
Phylogenetic analysis based on the nucleotide sequence of 3D gene. The tree was constructed by the neighbor-joining method with MEGA v.6.0. The numbers on the branches are bootstrap values (percent) from 1000 replicates. The three PSV strains isolated in this study are marked with a red dot, and the sequences of other representative PSVs are indicated as ‘GenBank accession number+PSV-1+Strain number+Country+Year’.

**Figure 7:**
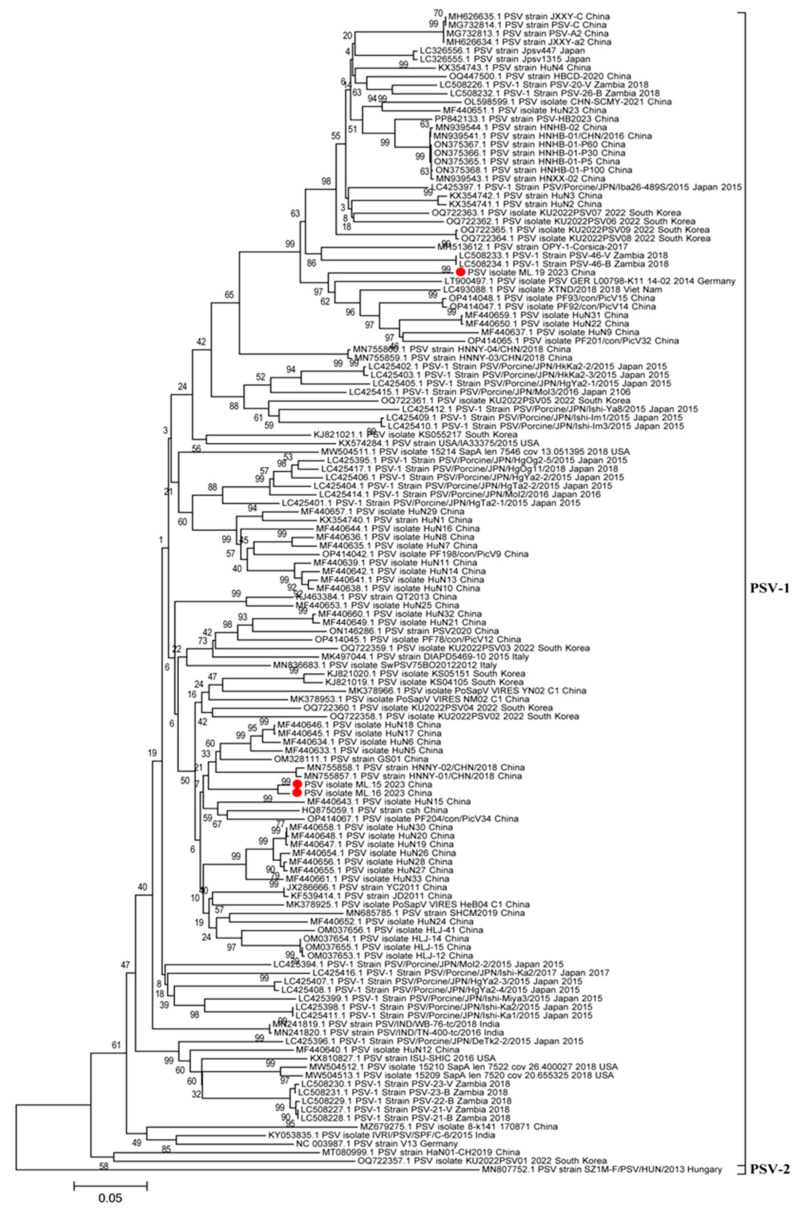
Phylogenetic analysis based on the nucleotide sequence of VP1 gene. The tree was constructed by the neighbor-joining method with MEGA v.6.0. The numbers on the branches are bootstrap values (percent) from 1000 replicates. The three PSV strains isolated in this study are marked with a red dot, and the sequences of other representative PSVs are indicated as ‘GenBank accession number+PSV-1+Strain number+Country+Year’.

To understand the phylogenetic relationships between the PSV strains isolated in this research and the other reference PSV strains, the complete-genome nucleotide sequences, VP1 gene, and 3D gene were used to construct phylogenetic trees. In the phylogenetic tree based on the genome sequences, different PSV strains were distributed into four clusters (cluster I to IV) (Figure 5). All of the Chinese PSV strains, including the three isolates included in this research, were in cluster I, while most of Japan strans and USA strains and partial South Korea strains were in cluster II; cluster III consisted of strains from France, Italy and Germany, and the India strains formed cluster IV (Figure 5). In the phylogenetic tree of the 3D gene, the Chinese PSV strains were in cluster I, and the PSV strains isolated from Japan, USA and South Korea strains were in cluster II, but the sequences from other countries were shuffled on the phylogenetic trees, and did not cluster closely (Figure 6). As for the VP1 gene, the phylogenetic topology was messier when compared with the whole genome and 3D gene and was unable to mirror the geographical origin of the strains (Figure 7). The sole PSV-2 strain SZ1M-F/PSV/HUN/2013, however, formed an independent branch from the PSV-1 strains, and the three PSV strains in this study clustered together in the PSV-1 group (Figure 7).

### 3.4. Recombination Analyses of the PSV Strains

**Figure 8:**
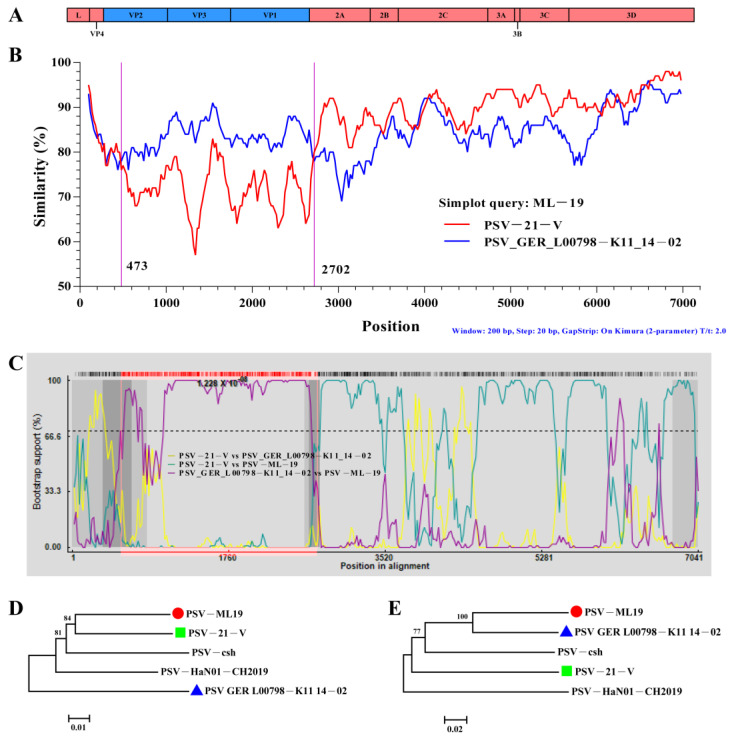
Genome recombination analyses of PSV-ML-19. (**A**) The genome schematic diagram of PSV: the red area indicates the region of origin from PSV-21-V, and the blue area indicates the region of origin from PSV GER L00798-K11 14-02. (**B**) Similarity plot analysis of the genome sequences of PSV-21-V (red curve), GER L00798-K11 14-02 (blue curve) and PSV-ML-19 as query sequences; the y-axis presents the percent identity with a window size of 200 bp and a step size of 20 bp. The vertical purple line indicates the potential recombination breakpoint. (**C**) The recombination breakpoint analysis of PSV-21-V vs. PSV GER L00798-K11 14-02 (yellow curve), PSV-21-V vs. PSV-ML-15 (light green curve), and PSV GER L00798-K11 14-02 vs. PSV-ML-15 (purple curve). (**D**) The phylogenetic tree constructed by the neighbor-joining method in MEGA 6.06 based on the concatenated sequences of the major parent. (**E**) The phylogenetic tree constructed by the neighbor-joining method in MEGA 6.06 based on the sequences of the minor parent.

To research the possible recombination events in the genomes of the three isolated PSV strains used in the present study, SimPlot (Version 3.5.1) and RDP4 (Version 4.101) software was used to analyze the recombination. The standard similarity plot analysis, which used the polyprotein gene of ML-19 as a separate query, revealed that ML-19 showed high nucleotide sequence similarity with PSV strain GER L00798-K11 14-02 (LT900497.1) in the sequences of VP1, VP2, and VP3, whereas the sequences of other genes (L, VP4, and 2A to 3D) were highly similar to the sequence of PSV strain PSV-21-V (LC508227.1) (Figure 8A). In order to identify potential breakpoints in the recombinant, we performed a bootscanning analysis using the RDP4 software (Version 4.101), and putative recombinant hotspots located in the VP2 and 2A gene regions (*p* ≤ 7.112 × 10^−10^) were identified using six different methods: RDP, Chimaera, BootScan, 3Seq, MaxChi, and SiScan (Figure 8B). Additionally, in the phylogenetic tree constructed based on the concatenated sequences both before and after the putative breakpoints (regions 1 to 530 and 2702 to 6872), ML-19 had the closest relationship with Zambian strain PSV-21-V (LC508227.1) (Figure 8C), while in the putative recombinant regions (positions between 530 and 2702), ML-19 and PSV-21-V (LC508227.1) were found in two different sublineages (Figure 8D). Collectively, these findings suggested that ML19 might be a recombined strain and may have emerged through genetic recombination between the major putative parent strain PSV-21-V and the minor putative parent GER L00798-K11 14-02.

## 4. Discussion

In summary, we isolated, identified, and sequenced three PSV strains PSV-ML-15, PSV-ML-16, and PSV-ML-19 from diarrheal samples sourced from pig farms in Yunnan Province in 2023, conducting comprehensive phylogenetic analyses of the isolated strains.

The isolation and culturing of PSV are usually performed on porcine kidney cell lines, such as PK-15, LLC-PK, and IBRS-2 [26], as well as swine testis (ST) samples and porcine intestinal epithelial cells (IPEC-J2), although these are used less frequently. PSV produces a distinctive CPE on PK-15, as exhibited by the enlarged, rounded, and gradually detaching cell. A previous study has reported that PSV can proliferate on human, baby hamster, and green monkey cell lines, such as 293T, BHK21, and Vero E6, which raises concerns regarding its host range potentially extending beyond swine; however, different susceptibilities to PSV may prove useful in the search for PSV receptors [3,12]. The PSV strains isolated in this study could not proliferate on Vero or BHK21, perhaps due to the different cell line sources and virulent strain types.

In 2016, Li Y et al. investigated a total of 185 fecal samples in Jiangxi Province, China; the results showed that 17.30% (32/185) were PSV/PEDV-double-positive [3]. Su M et al. investigated 543 diarrhea samples from 22 provinces in China between 2015 and 2018; the results indicate that the co-infection rate of PEDV among the PSV-positive samples was 43.33% [27]. Vilar MJ et al. analyzed 352 pig fecal samples from six different farms in Spain; the results here illustrated that the total prevalence of PSV-1 was 2.84% (10/3520), with all the piglets who tested positive for PSV-1 also testing positive for EV-G [28]. Stäubli T et al. studied the distribution of enteric viruses in Swiss pigs by testing 363 fecal, brain, and placental or abortion samples. Of the 76 fecal samples from healthy animals, PTV was detected in 47%, PSV-A in 51%, and EV-G in 70% [29], suggesting a high probability of PSV infection among healthy animals. Ham merschmitt ME et al. investigated the presence of PSV in a neurological disease outbreak which occurred on a swine farm in Southern Brazil. Among the 10 piglets’ postmortem examinations, 80% (8/10) of the piglets’ central nervous systems (CNSs) tested positive for PSV, while the detection rate of PTV was 100% [30], affirming that co-infection with PSV and PTV is very common in this survey. In this study, three isolates of PSV co-infected with PEDV, PTV, or EVG. Overall, PSV constantly co-infected with other diarrhea viruses, which is consistent with the results of this study.

Only the complete CDS was used to analyze sequence similarity as there were few complete-genome sequences that were submitted to the GenBank. On the other hand, a large volume of complete CDS sequences was available in GenBank. Furthermore, the 5′UTR and 3′UTR sequences were highly conserved between different PSV strains, and the length of the CDS (7000 nt) was about 93% of the whole genome (7500 nt). Therefore, we believe that the results of the complete CDS sequence analysis reflect the genetic evolution characteristics of the PSV strain.

VP1 has already been established as one of the outer capsid proteins of PSV and the C-terminal situated on the surface of virions, and has also been known to induce the production of host-neutralizing antibodies. The VP1 gene was the most variable gene among all twelve genes of the PSV strains [8]. In this study, we compared the VP1 genes of the isolated PSV strains with those of other PSV strains. We found that the VP1 gene of ML19 was 24 nt shorter than that of ML15 and ML16; correspondingly, the VP1 amino acid sequence was eight amino acids shorter (ATQTGFYP). It was noted that this deletion was also observed in the PSV strains isolated from China, Japan, South Korea, India, Vietnam, the USA, Zambia, and Germany, indicating that the deletion of the VP1 gene is not a result of it being an individual strain, but rather of it being a PSV VP1 gene. Further studies should be conducted to explore the causes of this deletion and its effects on the biological characteristics of PSV and its pathogenicity to the host.

In the present study, we conducted a genetic evolution analysis on a total of 136 sequences (133 reference sequences and the 3 sequences obtained in this research). The results were similar to those of previous studies, although some strains in the phylogenetic tree did not coincide with other geographical distributions. Most PSV strains showed a certain degree of geographical specificity, mainly being found clustered together as geographic branches in the topotypes of the Chinese strain and Japanese, South Korean, and European strains. We understand that the current research results do not reveal the authentic genetic evolution characteristics of PSV strains on a global scale, as more than half of the analyzed sequences (76/133) in this study were from Chinese strains, in addition to 24, 12, 3, and 1 being from Japanese, South Korean, Indian, and Vietnamese strains. Overall, 85.3% of all the analyzed strains were Asian, while sequences from other continents (Europe, America, Africa, etc.) only accounted for 14.7%. Therefore, it was impossible to accurately clarify the molecular genetic characteristics of PSV strains from other countries. According to the previous studies, PSV was prevalent in America [15], Europe [28,29], and Africa [17]. The global prevalence characteristics of PSV strains could be clarified further if more PSV sequences from these countries were submitted to the GenBank.

Genomic RNA recombination is a major driving force of RNA virus evolution [31], and recombination events were identified in several viruses within the family Picornaviridae (PSV), human enteroviruses (EV), the foot-and-mouth disease virus (FMDV), and EV-G [32,33,34,35]. The potential recombination sites in the VP2 and 2A genes of the ML19 strain isolated in this study were identified, and the recombination breakpoint in the 2A gene was consistent with multiple previous reports [32,36,37]. The recombination breakpoint in the VP2 gene was only recently reported by “Qiu-Yong Chen” in 2023 [8]. Therefore, according to the current study results, we inferred that the conjunction region of the VP1 and 2A genes was a major recombination region within the PSV genome, while the recombination frequency in VP2 was relatively low. Furthermore, we speculated that the reason for the frequent recombination observed within the conjunction region of the VP1 and 2A genes may be that the VP1 protein was located on the surface of the PSV virions, imposing great host selective pressure via the host immune system; as a result, different genetic variations (recombination, deletion, and insertion) were revealed to adapt to or evade the host immune system. It was interesting that the ML19 strain showed the highest mean similarity to the African PSV strains in terms of complete CDS and coding polyprotein sequences, while, for the single strain, ML-19 showed the highest similarity to Chinese strain HuN22. This indicates that ML19 and Zambian strains may share a common ancestor; however, the Chinese strains may have formed a specific topotype when the strains were prevalent in China. This observation is consistent with the evolution characteristics of the virus; many viruses are prevalent worldwide, while local epidemic strains may form endemic genomic topotypes [38,39].

PSV infection can induce piglet morbidity in the form of pneumonia, myelitis, or diarrhea. Li Y et al. orally inoculated 1-day-old piglets with 5 mL of PSV that then developed watery diarrhea; viral RNA was detected in the feces, all the alimentary tract organs, the tonsils, the inguinal lymph nodes, the spleen, the lungs, the cerebellum, the brain, and the bladder, but was not detected in the heart, liver, kidney, or spinal cord [3]. Liu Jiajia et al. injected 5 mL of PSV into 5-day-old piglets. The results showed high levels of the virus within the intestines and lungs, while the viral nucleic acids were not detected in the brain tissue, heart, liver, or kidney, suggesting that the mode of infection may influence the distribution of the virus. In the animal experiments of Lan D et al., 60-day-old pigs were given 4 mL of PSV orally; diarrhea and respiratory distress occurred at 2 days PI, and polioencephalomyelitis, presenting as ataxia and leg paralysis, occurred at 7 days PI [2]. The abovementioned results suggest that pathogenic differences between PSV strains may be related to the mode and dose of infection, the age of the experimental animals, and the differences between the experimental isolates. A decrease in the infected piglets’ temperature was detected through pathogenicity tests, suggesting a possible suppression of the body’s immune response. Lan D et al. illustrated that the PSV infection of IPEC-J2 cells can induce large amounts of type I interferon and can activate related interferon pathways [40]. Li Y et al. demonstrated that the PSV infection of ST cells promotes the expression of mRNA in IFN-β [3]. Yan Z. used qPCR, Western blot analysis, and IHC to detect proteins related to intestinal tight junctions in piglets infected with PSV, showing that PSV infection may downregulate the expression of intestinal-tight-junction-associated proteins, damage the integrity of the intestinal barrier, and cause intestinal injury. Kim DS et al. found that Korean SV-A strains could induce diarrhea and intestinal pathology in piglets but not in chicks, and could also spread to the bloodstream via the gut and disseminate to extra intestinal organs and tissues; this study’s results enhance our understanding of SV-A pathogenesis and the development of sapelovirus drugs and vaccines [27]. According to the phylogenetic analyses of the whole genome, the three PSV strains isolated in this research were in the same cluster (cluster 1), while the ML-15 and ML-16 strains showed long genetic distance with the ML-19. Therefore, understanding their pathogenicity and pathogenesis should guide future research.

## Figures and Tables

**Table 1:** Nucleotide and amino acid sequence identity (%) between PSVs isolated from Yunnan and reference PSV strains.

Isolate	Gene/Protein ^a^	Asia ^b^	America ^b^	Africa ^b^	Europe ^b^
**PSV15**	CDS (6996/2332)	**87.76** ± 1.85/**96.05 ±** 1.58	84.92 ± 0.97/94.46 ± 0.79	86.38 ± 0.46/94.58 ± 0.42	84.98 ± 0.98/94.10 ± 1.29
	L (252/84)	**88.37** ± 1.29/**96.42** ± 1.87	86.48 ± 1.06/95.20 ± 0	87.63 ± 0.46/95.07 ± 0.72	87.94 ± 0.63/96.40 ± 1.47
	VP4 (159/53)	79.59 ± 2.97/91.32 ± 2.58	81.46 ± 2.28/95.06 ± 3.80	**81.57** ± 2.72/**96.62** ± 4.01	76.26 ± 3.83/90.74 ± 5.21
	VP2 (714/238)	**85.39** ± 3.09/**96.12** ± 2.46	81.50 ± 3.19/94.14 ± 1.86	81.11 ± 1.87/93.33 ± 1.50	82.66 ± 2.30/94.66 ± 1.76
	VP3 (702/234)	**85.07** ± 3.62/**94.15** ± 3.90	79.78 ± 2.93/89.72 ± 3.19	78.72 ± 3.45/89.48 ± 2.03	83.56 ± 1.90/92.94 ± 2.85
	VP1 (879/293)	**81.27** ± 4.83/**88.32** ± 6.40	79.52 ± 2.46/87.52 ± 3.88	77.03 ± 2.28/82.78 ± 2.58	79.78 ± 5.67/85.82 ± 7.16
	2A(678/226)	**87.57** ± 3.54/**97.00** ± 2.64	85.14 ± 0.96/96.88 ± 0.53	86.74 ± 3.46/94.99 ± 3.23	81.00 ± 1.29/88.88 ± 1.04
	2B (315/105)	**87.93** ± 1.82/99.41 ± 0.84	86.76 ± 1.75/**99.80** ± 0.45	84.96 ± 0.65/99.56 ± 0.88	86.76 ± 0.59/97.84 ± 1.25
	2C (996/332)	89.24 ± 1.80/98.25 ± 2.09	85.26 ± 0.45/94.74 ± 0.13	**91.07** ± 0.56/**98.70** ± 0.30	87.90 ± 1.00/97.38 ± 1.51
	3A (300/100)	**89.30** ± 4.04/96.00 ± 2.09	86.16 ± 1.36/91.20 ± 0.84	86.04 ± 2.92/**96.89** ± 0.33	86.24 ± 5.46/95.40 ± 0.55
	3B (66/22)	88.87 ± 3.26/92.97 ± 3.94	86.92 ± 2.96/83.62 ± 4.07	**93.22** ± 2.03/**97.46** ± 3.32	87.22 ± 3.79/93.58 ± 4.07
	3C (546/182)	90.81 ± 1.52/98.61 ± 0.72	91.08 ± 0.31/98.54 ± 0.84	**91.10** ± 0.74/**98.67** ± 0.95	87.90 ± 1.15/98.20 ± 0.62
	3D (1389/463)	92.66 ± 1.93/**98.72** ± 0.75	90.04 ± 0.64/98.36 ± 0.68	**92.81** ± 0.40/98.69 ± 0.13	88.48 ± 0.94/97.44 ± 1.31
**PSV16**	CDS (6996/2332)	**87.69** ± 1.86/**95.81** ± 1.57	84.92 ± 1.02/94.46 ± 0.83	86.34 ± 0.54/94.41 ± 0.45	84.92 ± 0.93/93.94 ± 1.30
	L (252/84)	**89.13** ± 1.30/97.54 ± 1.81	86.48 ± 1.05/95.20 ± 0	88.40 ± 0.49/96.27 ± 0.72	88.72 ± 0.66/**97.60** ± 1.47
	VP4 (159/53)	80.21 ± 2.97/93.19 ± 2.59	81.46 ± 2.50/95.06 ± 4.16	**82.20** ± 2.71/**94.72** ± 4.01	76.86 ± 3.89/89.00 ± 5.12
	VP2 (714/238)	**84.75** ± 3.07/**95.31** ± 2.46	81.50 ± 3.26/94.14 ± 1.83	80.66 ± 1.79/92.47 ± 1.55	81.92 ± 2.35/93.78 ± 1.75
	VP3 (702/234)	**85.10** ± 3.71/**94.15** ± 3.90	79.78 ± 2.90/89.72 ± 3.19	78.99 ± 3.13/89.48 ± 2.03	83.52 ± 1.93/92.94 ± 2.85
	VP1 (879/293)	**81.31** ± 4.72/**88.20** ± 6.50	79.52 ± 2.36/87.52 ± 3.67	77.31 ± 2.28/82.67 ± 2.81	79.88 ± 5.25/85.56 ± 7.35
	2A(678/226)	**87.12** ± 3.56/95.96 ± 2.40	85.14 ± 0.95/**96.88** ± 0.53	86.31 ± 3.81/94.19 ± 3.01	80.68 ± 1.00/88.36 ± 1.04
	2B (315/105)	**87.62** ± 1.81/98.42 ± 0.81	86.76 ± 1.83/**99.80** ± 0.45	84.78 ± 0.47/98.58 ± 0.84	86.22 ± 0.72/96.88 ± 1.22
	2C (996/332)	89.39 ± 1.81/98.25 ± 2.09	85.26 ± 0.51/94.74 ± 0.13	**90.92** ± 0.64/**98.70** ± 0.30	88.08 ± 0.83/97.38 ± 1.51
	3A (300/100)	**89.48** ± 4.15/96.00 ± 2.09	86.16 ± 1.09/91.20 ± 0.84	86.12 ± 2.92/**96.89** ± 0.33	86.50 ± 5.21/95.40 ± 0.55
	3B (66/22)	88.87 ± 3.26/92.97 ± 3.94	86.92 ± 2.96/83.62 ± 4.07	**93.22** ± 2.03/**97.46** ± 3.32	87.22 ± 3.79/93.58 ± 4.07
	3C (546/182)	90.84 ± 1.55/98.08 ± 0.71	**91.08** ± 0.29/**98.54** ± 0.45	90.90 ± 0.74/98.17 ± 0.95	87.80 ± 1.06/97.68 ± 0.59
	3D (1389/463)	92.51 ± 1.92/98.53 ± 0.75	90.04 ± 0.64/98.36 ± 0.75	**92.76** ± 0.43/**98.69** ± 0.13	88.38 ± 1.03/97.40 ± 1.28
**PSV19**	CDS (6996/2332)	86.92 ± 1.46/95.14 ± 1.24	84.50 ± 0.42/93.36 ± 0.21	**87.07** ± 0.72/**95.16** ± 0.92	84.26 ± 1.20/94.00 ± 1.52
	L (252/84)	91.18 ± 1.79/**97.14** ± 1.65	**92.48** ± 0.95/96.40 ± 0	91.07 ± 0.72/96.27 ± 0.72	88.66 ± 1.18/96.16 ± 2.31
	VP4 (159/53)	**84.96** ± 2.36/**96.00** ± 1.77	83.48 ± 1.89/93.16 ± 3.18	81.88 ± 1.95/94.09 ± 2.00	74.1 ± 2.35/85.82 ± 4.20
	VP2 (714/238)	**84.26** ± 2.82/94.45 ± 2.14	80.96 ± 2.54/93.56 ± 0.76	80.80 ± 2.49/93.71 ± 2.05	82.22 ± 1.69/**94.98** ± 1.43
	VP3 (702/234)	**82.84** ± 1.74/92.73 ± 2.08	79.88 ± 1.87/91.04 ± 0.95	80.81 ± 4.64/91.83 ± 3.37	82.66 ± 3.31/**93.62** ± 3.37
	VP1 (855/285)	76.30 ± 3.58/81.65 ± 5.32	73.64 ± 2.02/78.20 ± 0.79	**79.01** ± 2.74/**83.72** ± 5.37	77.72 ± 4.75/83.96 ± 7.55
	2A(678/226)	**87.67** ± 3.34/96.99 ± 2.67	85.06 ± 0.73/**97.12** ± 0.71	86.99 ± 2.87/94.50 ± 3.47	81.50 ± 1.07/89.52 ± 1.08
	2B (315/105)	89.54 ± 1.94/99.41 ± 0.84	88.52 ± 1.10/**99.80** ± 0.45	**89.59** ± 0.86/99.56 ± 0.88	85.92 ± 0.68/97.84 ± 1.25
	2C (996/332)	88.16 ± 2.06/97.87 ± 2.16	84.62 ± 0.56/94.14 ± 0.13/	**89.09** ± 0.64/**98.60** ± 0.21	86.42 ± 0.61/96.90 ± 1.25
	3A (300/100)	**89.46** ± 3.60/96.88 ± 2.55	85.88 ± 1.63/89.20 ± 0.84	85.82 ± 2.91/**98.22** ± 1.20	86.24 ± 6.03/95.60 ± 0.55
	3B (66/22)	88.23 ± 4.01/96.23 ± 3.57	86.32 ± 3.04/88.12 ± 4.07	**94.23** ± 2.78/**96.93** ± 2.30	84.80 ± 1.50/89.08 ± 4.07
	3C (546/182)	90.51 ± 1.37/98.94 ± 0.56	90.70 ± 1.20/98.66 ± 0.62	**91.74** ± 0.80/**99.16** ± 0.49	87.88 ± 0.58/98.86 ± 0.55
	3D (1389/463)	92.54 ± 1.36/99.06 ± 0.68	90.88 ± 0.56/98.60 ± 0.66	**93.16** ± 0.49/**99.17** ± 0.22	88.34 ± 0.95/97.82 ± 1.18

^a^ The number in the brackets is the length of the gene or the deduced protein; ^b^ The identity is represented as mean and SD, and the bold face numbers depict the highest identity.

## Data Availability

The complete-genome sequences of PSV-ML-15, PSV-ML-16, PSV-ML-19, obtained in this study have been deposited in the GenBank under the accession numbers PV009944, PV009945, PV009946, respectively.

## Supplementary Materials

The following supporting information can be downloaded at: www.mdpi.com/article/10.3390/v17040505/s1, Table S1: Nucleotide identity; Table S2: Amino acid identity.
